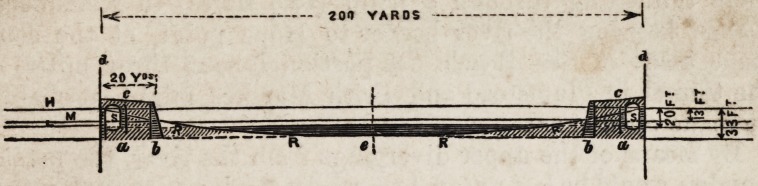# Scheme for the Drainage of London

**Published:** 1858-04

**Authors:** J. E. Huxley


### A SCHEME FOR THE DRAINAGE OF LONDON.

By J. E. HUXLEY, M.D.

THE following scheme is intended to combine the embankment of the Thames, on both sides, throughout the Metropolis, with the formation of main sewer canals, the contents of which shall be subject to a tidal flushing towards the mouth of the river, twice daily, from flood tide to two-thirds ebb.

All along the Thames, from Chelsea and Battersea to London Bridge, on both sides, the sewer canals, contained in the embankments, should be formed. At the necessary distance from the high water shores, and between these and the water, the outer sewer walls should be sunk and built. The depth of the canals should be 3 feet below the lowest ebb of the stream ; their height 4 feet below the soil and pavement of the embankments. Taking the rise of the Thames, at London Bridge, at 20 feet, the canals would thus be 25 feet in depth, and the top of the embankments 6 feet above high water. The breadth of the canals might be determined according to the area required,—say 20 feet, which would give a sectional area of 500 square feet. The height *only* of the canals might diminish somewhat from London Bridge upwards. The embankments should extend on each side along the river from Chelsea to London Bridge, or from such a point westward as shall fully include the western suburbs ; a width of 20 yards on each side, measuring across the bed from the high-water shore, being taken for the embankments. (See diagram.) As the river is nowhere, between the points named, less than 200 yards in width, the loss to the river bed (if real loss at all) would not exceed one-fifth part at the most ; and that fifth would be that part of the shore the least covered by water, and the least wholesome when exposed to the sun and air. (The "mud-larks" would want compensation for the loss of vested interests?)

The canals should be continued, *as covered canals merely,* such a distance up the river as would be necessary to prevent tidal reflux of their contents, and consequent mixture with the stream. The canals, at both ends, should be open, in order, at the upper, to admit the waters of the river, which are to form the carrying and flushing power; and, at the lower end, for the gradual discharge of the contents into the estuary at Sea Reach with the ebb of the tides. The waters of the river would enter the upper mouths of the canals, whenever the level was more than 7 feet above low water. The admission of the rising tides by the lower mouths would be the means of dislodging, stirring up, and keeping solid contents from subsidence, and ready to descend with every turn of the tide. All London surface-water, as well as sewage, should be allowed to enter the canals, both as an assistance to the movement of their contents, and a means of frequently lessening the amount of the river water abstracted by the canals from the purposes of navigation.

The fall in these canals should be only that of the river itself.

*Course.* After arriving at, or near to London Bridge, the north canal should leave the river edge (the embankment to cease) and take a subterranean course direct for Blackwall, clear of the Isle of Dogs and West India Docks, by a tunnel of from three to four miles long; then it should take the river border again, and pursue it to Rainham Creek (about eight miles); thence by direct subterranean tunnel (through a point, two miles) to near Purfleet, when it would meet the river again, and follow it to near West Thurrock; thence it would tunnel again for two miles (through a point) regain the river, and follow it to Gray's Thurrock; thence it would tunnel again (east-north-east for about six miles) to its final termination at Sea Reach.

The last portion, between Gray's Thurrock and Sea Reach, as also the two miles at Rainham Creek, might, from the nature of the districts, be open canal.

The south canal should leave the river edge (and the embankment cease) at London Bridge, and take a subterranean course of about twelve miles, passing south of Greenwich,

Woolwich, and of Plumstead Marshes, and regaining the river just below Erith; continuing along the river to Greenhithe, there tunnelling through a point (two miles) to Northfleet; thence keeping the river border to Hope point, at the commencement of Sea Reach. A portion (about three miles) of the tunnel in Plumstead and Erith Marshes might, probably, be made open canal.

By means of the upper divergings from the river, the port of London would be left untouched, whilst the lower tunnels would obviate sharp bends in the river, which would offer obstacles if followed in the course of the canals. Occasional portions as open canal, where there was no sanitary objection, would be a safeguard to the works in case of extraordinary tides and floods. Twice a day the contents of the canals would be ebbing with the tide towards the sea; and also flowing twice in the opposite direction, but each time, from the accumulation of back water, falling short of the last point of departure.

*River above Chelsea.* Chelsea and Battersea should be the highest points drained *directly* into the canals; above these, the canals should be continued as unbroken tubes up stream, far enough to prevent the tides from driving back the contents, until *sewage* issue from the open mouths. But places on the river, situated higher up, and now draining into the Thames, should discontinue that practice, and be allowed (compelled?) to carry an independent main sewer, on each side, to receive successively the sewage from places on its course, and convey it to Chelsea and Battersea, there to be discharged into the metropolitan canals by a junction on the land side therewith.

*Saving the Sewage.* The question of saving and utilising the valuable contents of the canals ought not, in the first place, to be allowed to complicate and retard a work of such vital importance as the drainage of London; because the art of dealing with refuse matters on a large scale is not yet sufficiently advanced. At those points on the canals where it is proposed to make them open to the air (Rainham Creek, Gray's Thurrock, Plumstead Marshes), the required accessibility would be provided to any future scheme proposing to intercept sewage matter, and manufacture from it manure for the land.

*General Considerations.* Either the Thames must be used as the carrying power to rid London of its enormous sewage, or it must not. To resign it seems impossible; and it is, as all rivers are, the natural line of drainage. It is therefore desirable to derive its good offices in such a way as to preserve the main stream unpolluted, on behalf of navigation and of health. A river having such very widely different offices to perform, must be divided in order to keep the performances separate. The depth of the proposed canals is put at the difference between the lowest ebb and the highest flow, with so much added as may give security against damage from extraordinary tides, and from floods from the land. The loss in width of river at London would be more than compensated by the retentive form given by the artificial banks, by their greater regularity of figure, and their directness from point to point.

Engineering difficulties may be safely committed to the enterprising talent of the age. The tributaries Lea and Roding on the left bank, Ravensbourne and Darent on the right, may be some of such difficulties. But the authors of the *Report on Metropolitan Drainage*, with their proposed outfalls at Mucking and Higham Creek, must be already prepared to deal with these.

*Cost.* Greatness of cost is about the last consideration which ought to deter from any scheme embracing the power really to do the work. The money would be forthcoming, if, by Act of Parliament, a "Metropolitan Drainage Stock" could be created, paying a good rate of interest secured upon rates. Thus a new stock for permanent investment would be created, secured upon the existence of London ; and, therefore, not less safe than the 3 per cents., but more profitable to holders. If five millions sterling were required and spent, 5 per cent. thereon would not exceed 2*s*. per head per annum on the population of London. The amount expended, so that it be well spent, is therefore of no consequence.

One advantage of this scheme, which will be generally appreciated, is the preservation of all existing minor systems of house and street drainage in their present natural course towards the Thames. Look at the annoyance to persons in altering these ; whilst any scheme, not availing itself of the course of the river, must devote half its outlay to altering what now exists ! Places below London, on the river, should be allowed to embank over, and drain into, the metropolitan canals.

## Figures and Tables

**Figure f1:**